# Recent developments in one-pot stepwise synthesis (OPSS) of small molecules

**DOI:** 10.1016/j.isci.2022.105005

**Published:** 2022-08-27

**Authors:** Xiaoming Ma, Wei Zhang

**Affiliations:** 1 School of Pharmacy, Changzhou University, Jiangsu 213164, China; 2 Department of Chemistry, University of Massachusetts Boston, MA 02125, USA

**Keywords:** Chemistry, Organic chemistry, Green chemistry

## Abstract

One-pot synthesis is an active topic in organic chemistry due to its intrinsic advantages of simple operation, high mass efficiency, low cost, and less amount of waste disposal. Among three kinds of one-pot syntheses, 1) cascade reactions, 2) multicomponent reactions (MCRs), and 3) one-pot stepwise synthesis (OPSS), OPSS could be more flexible and practical since it is carried out stepwisely and have variable reaction conditions for different steps. This perspective article uses selected examples to highlight the recent development in OPSS involving cyclization, cycloaddition, rearrangement, and catalytic reactions for the synthesis of heterocyclic scaffolds, asymmetric molecules, natural products, and bioactive compounds.

## Graphical abstract

**Figure:**
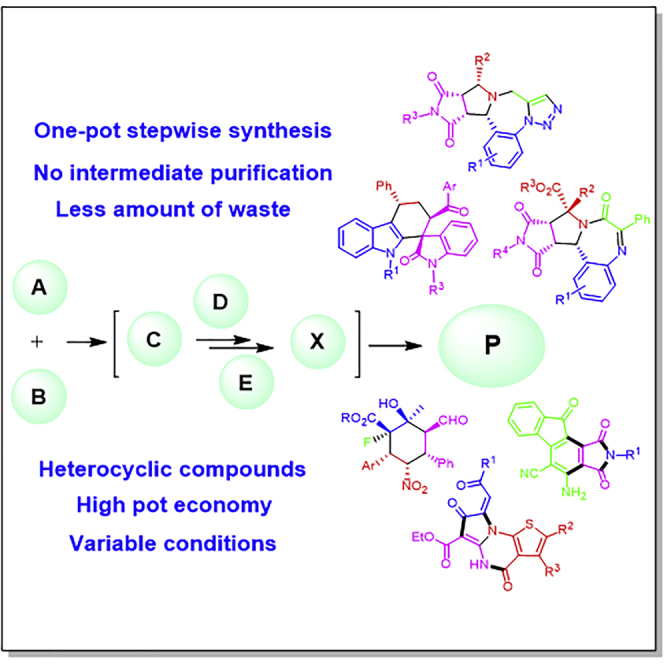

## Introduction

Synthetic chemistry poses constant demands on the development of more efficient and greener techniques to make the reaction processes to be high mass efficiency, minimal effort on intermediates isolation, less energy consumption, and with reduced amount of waste (Clark and Macquarrie, 2002; Anastas and Eghbali, 2010; Zhang and Cue, 2018). Performing organic reactions in single vessel (one-pot) is a good approach for high pot, atom, and step economy (PASE) (Trost, 2002; Wender, 2013; Clarke et al., 2007; Hayashi, 2016) and favorable in green metrics analysis such as E-factor and C factor (Constable and Jiménez-González, 2018; Sheldon, 2018; McElroy et al. 2015). The one-pot synthesis can be carried in three different ways: domino or cascade reactions (Tietze et al., 2006), multicomponent reactions (MCRs) (Zhu et al., 2014), and one-pot stepwise synthesis (OPSS) (Zhang and Yi, 2019). Examples on quantitative green metrics analysis could be found for MCR (Abou-Shehada et al., 2017) and OPSS (Climent et al., 2010).

**Scheme 1:**
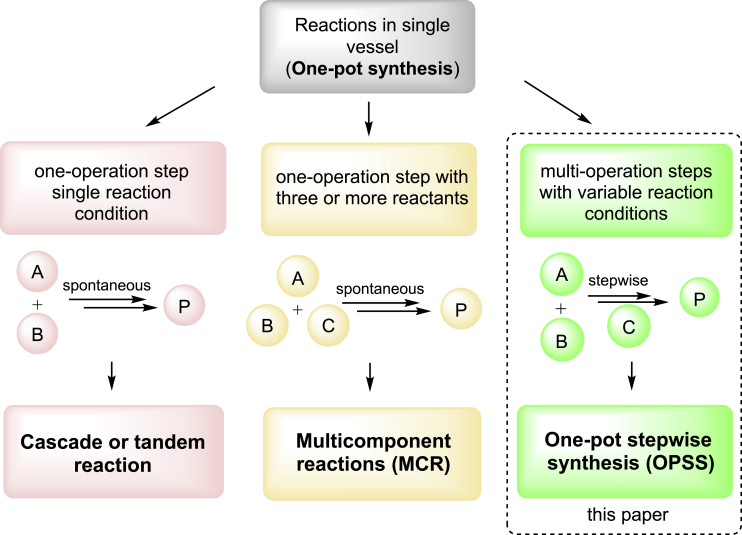
Conceptual figure of three one-pot reactions

Three one-pot reactions are different from their operation procedures (Scheme 1). A cascade reaction (also known as domino or tandem reaction) is a single-operation reaction but involving sequential chemical transformations. Once the reaction started, no additional reactants, reagents, or catalysts are introduced to the reaction system (Snyder and Schaumann, 2016; Pellissier 2020a). Total synthesis of (+/−) hirsutene via cascade radical cyclization is a good example (Curran and Rakiewicz, 1985). An MCR is also a single-operation reaction but has three or more reactants. The Ugi, Biginelli, Petasis, and Groebke-Blackburn-Bienayme are the well-known MCRs (Heravi and Zadsirjan, 2020). An OPSS has multi-operation steps, which allows reactants, reagents, and catalysts introduced stepwisely. The reaction conditions could be changed at each step. Solid-phase peptide synthesis (Merrifield, 1986), organic synthesis using solid-supported reagents and scavengers (Ley et al., 2000), polymer synthesis (Espeel and Du Prez, 2015), and synthesis of some biomolecules (Sui et al., 2021) are good examples of OPSS. Many biocatalysis-based OPSS have been reported in literature (Bruggink et al., 2003; Riva and Fessner, 2014; Groger and Hummel, 2014; Denard et al., 2013). This paper only covers solution-phase OPSS of heterocyclic small molecules with biological interests.

It is worth noting that “multistep” in this paper refers to *operation step*, not the *chemical transformation step* (Broadwater et al., 2005). Mislabeling of these three different kinds of one-pot reactions could be found in literature, which are caused by lack of clear differentiation on the operation step and the chemical transformation step. Defining the one-pot reactions based on the operation procedure shown in Scheme 1 could avoid following potential misnomers:

1) a single-operation reaction involving one reactant (intramolecular) or two reactants (intermolecular) with sequential chemical transformations should be called a cascade reaction instead of a multistep reaction;
2) a single-operation reaction with three or more components should be called MCR instead of a one-pot reaction;
3) a one-pot reaction with multi-operation steps should be called OPSS instead of a cascade reaction;
4) a one-pot synthesis with three or more components in multistep should be called OPSS instead of MCR.

**Figure 1:**
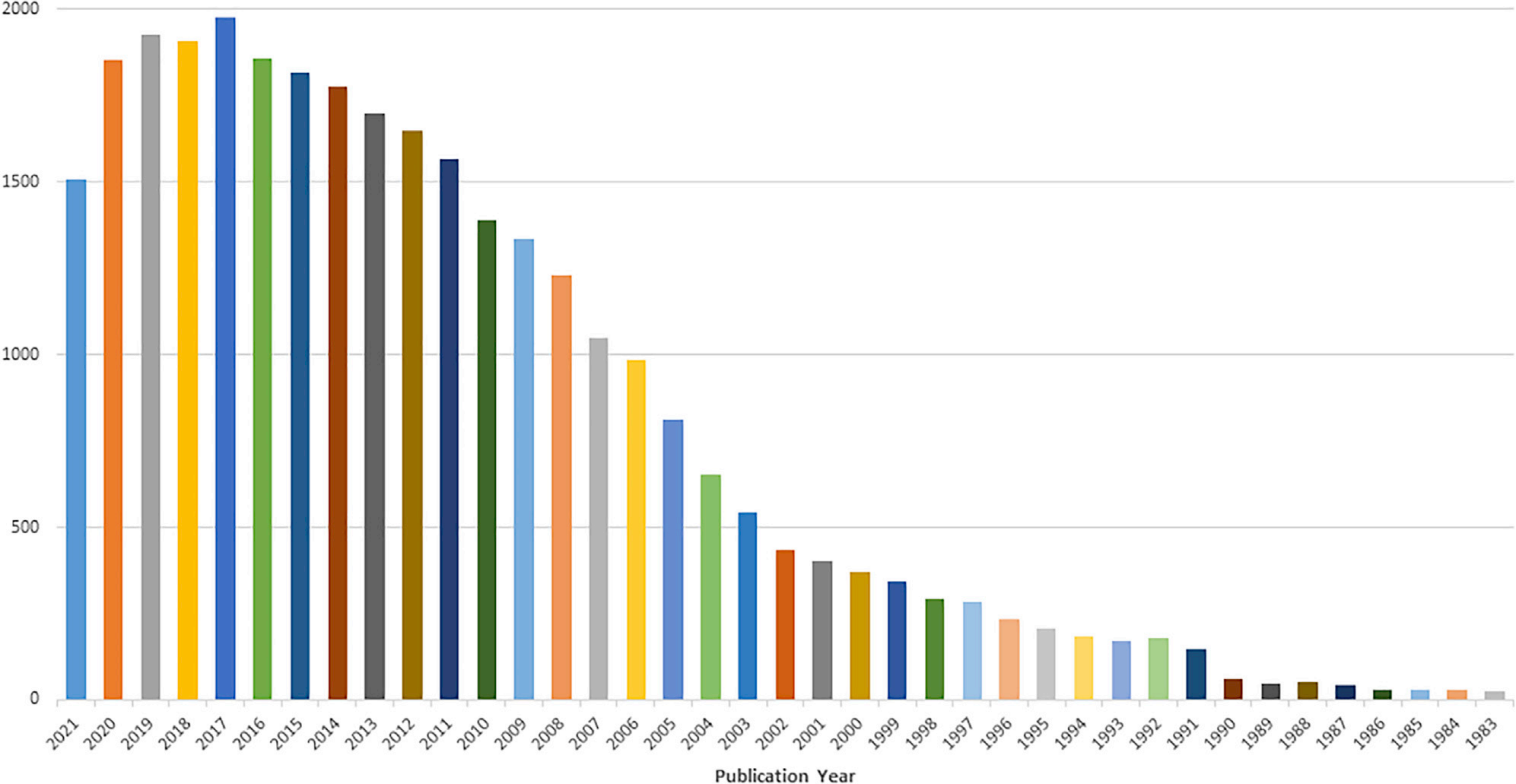
Distribution of “one-pot synthesis” papers by year

**Figure 2:**
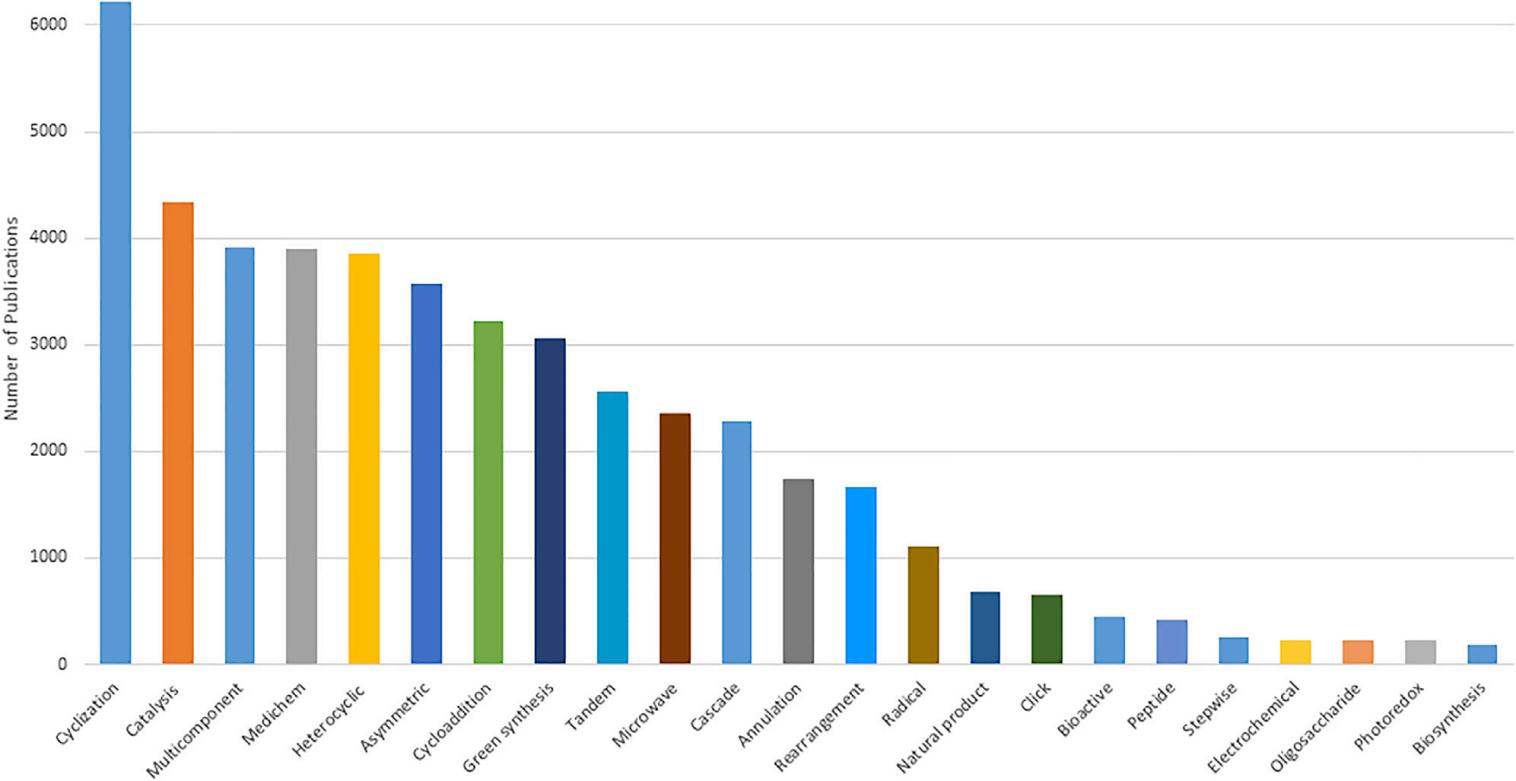
Distribution of “one-pot synthesis” papers by topic

One-pot synthesis has been an active topic for two decades. A Web of Science search on “one-pot synthesis” (for chemistry organic) gave 31,195 hits (available at: https://clarivate.libguides.com/webofscienceplatform/alldb, accessed on Jan. 17, 2022). As shown in Figure 1, there was a steady increase on the number of papers from early 1990s and peaked at 2017. In last ten years, more than 1,500 papers on this topic published each year. The concept and practice of one-pot synthesis have been well integrated to different organic transformations such as cyclization, cycloaddition, rearrangement, catalysis, radical, click, microwave, electrochemical and photoredox reactions in the synthesis of heterocyclic compounds, drug molecules, biomolecules, and natural products (Figure 2). The number of publications on these three kinds of one-pot synthesis is quite different, MCRs 3,907, cascade reactions 2,287, but only 254 for stepwise reactions (173 if search on multistep reactions). MCRs (Cioc et al., 2014; Domling et al., 2012) and cascade reactions (Ardkhean et al., 2016), especially the radical cascade reactions (Huang et al., 2019; Zhang and Studer, 2015), have been well documented. However, other than a conceptual paper (Broadwater et al., 2005), no dedicated review articles on OPSS of small molecules could be found in literature. Some OPSS appeared in the review articles which are mainly for cascade-type synthesis (Zheng and Hua 2020; Martínez et al., 2021; Hayashi, 2021).

The numbers of MCRs are limited and not so easy to discover new MCRs. The cascade reactions are generally good for carrying out similar kind of transformations (such as radical reactions) in one-pot fashion. OPSS is more flexible and practical because the reactions are stepwise and conditions are variable. OPSS has more parameters for reaction condition optimization, good for exploring the scope of substrates, suitable for scale-up reactions, and capable to integrate different kinds of reactions. Other than developing new OPSS, there is a significant amount of recent effort on reengineering the known multistep synthesis to be one-pot synthesis. There are several general practices in the development of OPSS:

1) maximizing the conversion of the initial reaction to minimize the impact of side-products to the later reactions;
2) avoiding harsh reaction conditions such as using strong acid/base which may not be compatible to other transformations;
3) switching solvents and changing reaction temperature at different steps to address reactant solubility and reactivity issues;
4) balancing functional group tolerance, substrate scope, and reaction selectivity throughout the whole reaction process;
5) generating unstable and highly reactive reactants *in situ* and integrating to the reaction sequence;
6) performing complicated and/or sensitive reactions (such as MCR or transition metal-catalyzed reactions) at the beginning of the synthesis to minimize the impact of side-products from the previous steps.

This perspective article highlights the recent development of solution-phase OPSS of small molecules. Examples are selected from the synthesis of heterocyclic molecules, privileged structures, and natural products with biological interests. They are presented in this paper based on the chemical transformations including cyclization, cycloaddition, rearrangement, multicomponent reactions, and metal-catalysis or organocatalysis. Solid-phase or solid-supported OPSS (Ley et al., 2000), biocatalytic OPSS (Bruggink et al., 2003; Riva and Fessner, 2014), and flow chemistry-based OPSS (Vaccaro, 2017) are not covered in his paper.

### Cyclization reactions

As shown in Figure 2, even cyclization reactions, including ionic, radical, and metal-catalyzed coupling have more than 6000 papers in one-pot synthesis. However, OPSS-related cyclization reactions are much less (<70) which means most one-pot cyclization reactions are cascade or tandem reactions instead of MCRs or OPSS (Tietze et al., 2006; Zhang and Yi, 2019).

**Scheme 2:**
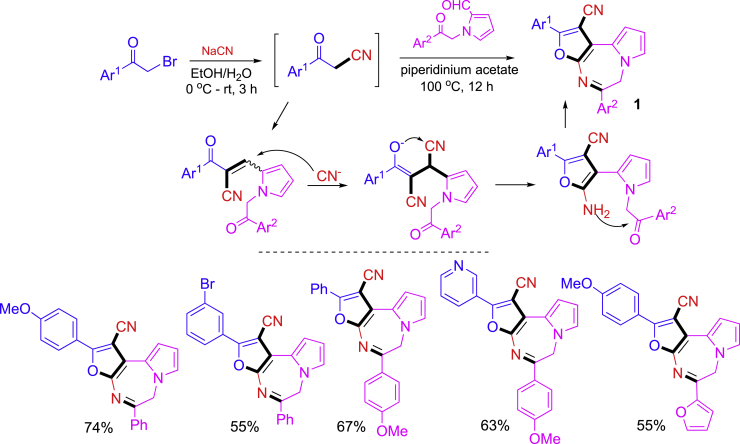
A two-step synthesis of diazepine-fused tricyclic compounds

Shown in Scheme 2 is a one-pot two operation-step sequence involving cyano substitution of *α*-bromo ketones, aldol condensation, furan formation, and intramolecular amination for the synthesis of tricyclic compounds **1** (Yoon et al., 2020). The phenacyl bromide was first reacted with NaCN at 0°C to room temp for 3 h, then a pyrrolyl aldehyde and piperidinium acetate were added and the reaction mixture was heated at 100°C for 12 h to afford diazepine-fused tricyclic compounds. In this synthesis, the first step is only for Br/CN exchange, the remaining transformations including double nucleophilic cyclization were completed in the second step of introducing aldehydes.

**Scheme 3:**
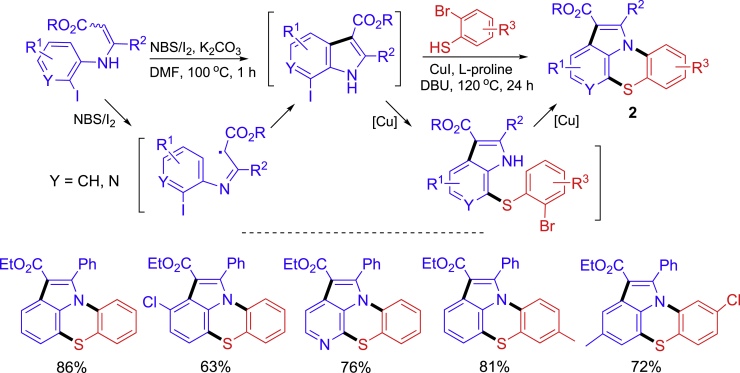
A two-step synthesis of thiazino[2,3,4-*hi*]indoles

A one-pot two-step synthesis of thiazino[2,3,4-*hi*]indoles **2** from *o*-haloaryl enamines and *o*-bromothiophenols is shown in Scheme 3 (Mao et al., 2017). In this reaction sequence, NBS/I_2_-promoted radical cyclization of enamines produced 7-iodoindole-type intermediates which then reacted with bromothiophenols for Cu-catalyzed intermolecular C-S coupling and intramolecular C-N coupling to afford the tetracyclic products. The iodo group is more reactive than the bromo group, so the C-S coupling occurred prior to the C-N coupling. This project is a good example of integration radical cyclization and metal-catalyzed inter- and intramolecular coupling reactions in one-pot for making a novel heterocyclic scaffold.

**Scheme 4:**
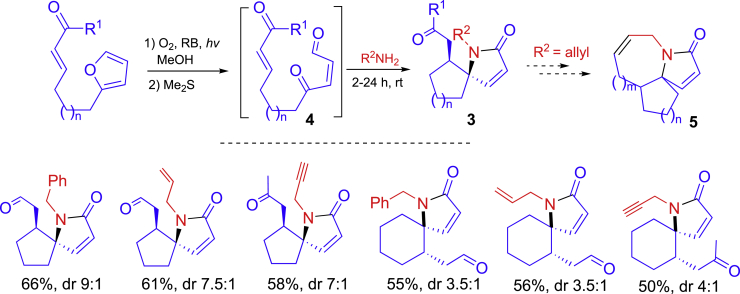
A three-step synthesis of 1-azaspirocyclic scaffolds **3**

A stepwise synthesis of 1-azaspirocyclic scaffolds **3** has been developed from the reaction of furyl aldehydes (Scheme 4) (Hoxha et al., 2021). The furyl aldehydes with different length of linkers (n = 1 or 2) underwent photooxygenation on the furan ring with O_2_ and then Me_2_S. The resulted tricarbonyl compounds **4** reacted with primary amines for [5 + 1] annulation to afford 1-azaspiro[4.4]nonanes or 1-azaspiro[4.5]decanes **3**. The products could be converted to dienes for ring-close metathesis in the synthesis of tricyclic scaffolds **5** of natural alkaloid compounds. This is a good example of integration green techniques (one-pot, O_2_, photoreaction, and MeOH solvent) for organic synthesis.

**Scheme 5:**
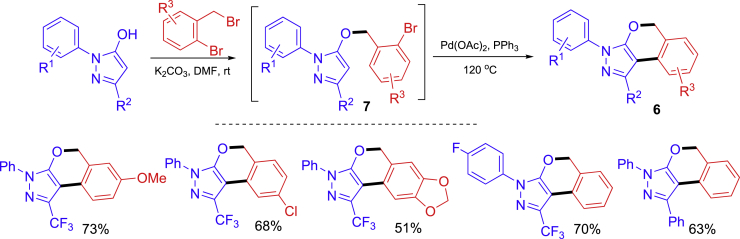
A two-step synthesis of isochromene-fused pyrazoles

A stepwise nucleophilic substitution and Pd-catalyzed C_sp2_-C_sp2_ biaryl coupling reaction sequence has been developed in the synthesis of isochromene-fused pyrazoles **6** (Scheme 5) (Nikolić et al., 2020). The reaction of hydroxypyrazoles and benzyl bromides gave intermediate **7** which were used for Pd-catalyzed C–H arylation to assemble the 6-membered ring of the tricyclic products. Same solvent (DMF) and base (K_2_CO_3_) were used for the two reaction steps. The second step was performed by simply adding Pd-catalyst to the reaction mixture and increases the reaction temperature.

Other than above mentioned cases, more examples could be found in the synthesis of heterocycles such as indazol-3-ones (Liu et al., 2019), benzonaphthyridines (Kumar et al., 2017), pyrazolones (Howard et al., 2017), and pyrazolines (Ganesan et al., 2020).

### Cycloaddition reactions

A cycloaddition is more powerful than a cyclization in the building up of molecular diversity and complexity (Gabriele et al., 2019). Because it involves multiple reaction centers and with high stereochemistry requirements, integration of cycloadditions in one-pot reactions could be more complicated than cyclizations, especially in the performing of consecutive cycloadditions (Sears and Boger, 2016).

**Scheme 6:**
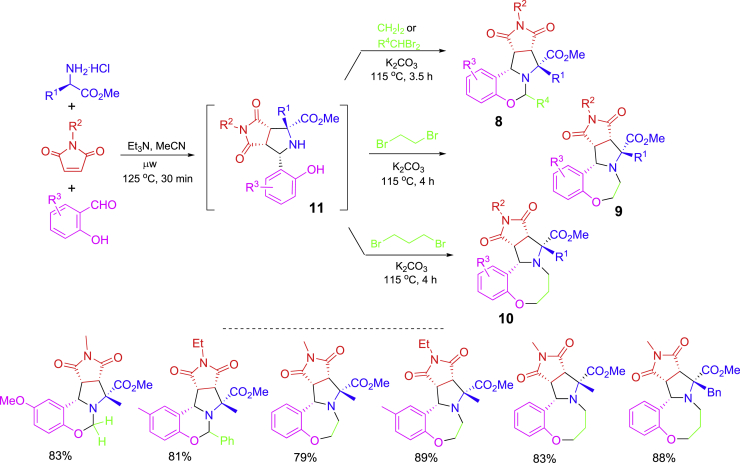
A two-step modular synthesis of three different ring scaffolds

A two-step protocol involving [3 + 2] cycloaddition and nucleophilic annulation has been developed for modular synthesis dihydrobenzoxazines **8**, tetrahydrobenzoxazepines **9**, and tetrahydrobenzoxazocines **10** (Scheme 6) (Muthengi et al., 2018). Azomethine ylides generated *in situ* from aminoesters and benzaldehydes underwent 1,3-dipolar cycloaddition with maleimides to give [3 + 2] adducts **11** which were used as common intermediates for three different nucleophilic [4 + n] annulation reactions to form 6-, 7-, and 8-membered ring-fused tetracyclic compounds, respectively. Et_3_N was used as a base for the first step cycloaddition under microwave heating at 125°C for 30 min K_2_CO_3_ was added to the reaction mixture to promote the nucleophilic [4 + n] annulation under conventional heating at 125°C for 3.5–4 h.

**Scheme 7:**
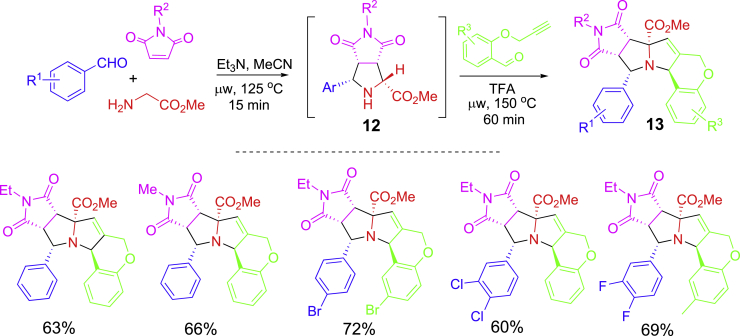
One-pot inter- and intramolecular [3 + 2] cycloaddition for condensed ring system

Another [3 + 2] cycloaddition-initiated one-pot synthesis is shown in Scheme 7 (Zhang et al., 2018). In this case, *α*-unsubstituted amino esters were used in the initial [3 + 2] cycloadditions with benzaldehydes and maleimides. The resulted proline ester derivatives **12** which have *α*-proton could be used for intramolecular [3 + 2] cycloadditions with propargyl benzaldehydes to assemble a condensed ring system **13** in a diastereoselective manner. Both reaction steps were performed under microwave heating. Et_3_N was used to promote the first cycloaddition, while TFA was added to improve the conversion for the second cycloaddition. It is a highly operational efficient and mass efficient synthesis, only 2 equiv of water was generated as a byproduct.

**Scheme 8:**
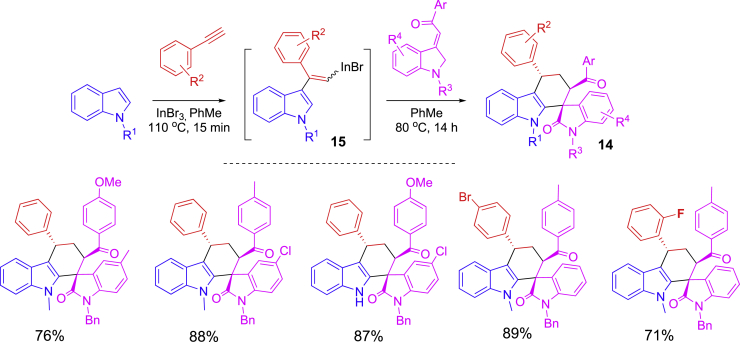
Two-step Diels-Alder reaction for spiro heterocyclic compounds

The Diels-Alder reaction is one of the most popular cycloaddition reactions. It has been integrated into a one-pot process involving the formation of dienes and sequential [4 + 2] cycloaddition in the synthesis of a spiro heterocyclic scaffold **14** containing indolinone and indoline moieties (Scheme 8) (Wang et al., 2021). The first step reaction was InBr_3_-catalyzed coupling of indoles with phenylacetylenes to form 2-alkenylindoles **15** which then served as dienes to react with olefinic isatin for [4 + 2] cycloaddition to form spiro compounds **14** diastereoselectively. Lewis acid InBr_3_ catalyzed both reactions and also contributed to the transition states to control the diastereoselectivity, which is a key factor to the success of this one-pot synthesis.

### Rearrangement reactions

As a mass efficient chemical transformation, the rearrangement reaction reorganizes the molecular structure without releasing byproducts (Jones et al., 2014). The reactions are commonly performed under relatively simple catalytic or heating conditions.

**Scheme 9:**
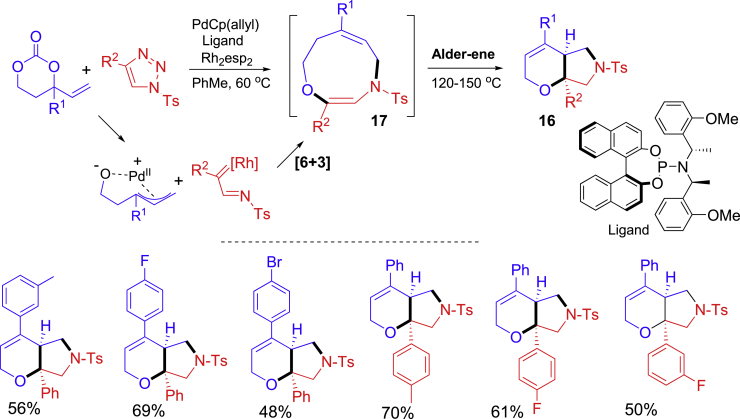
One-pot synthesis involving transannular Alder-ene rearrangement

Highlighted in Scheme 9 is a transition metal-catalyzed [6 + 3] cycloaddition followed by transannular Alder-ene rearrangement to form bicyclic hexahydropyranopyrroles **16** (Lee et al., 2021). The [6 + 3] cycloaddition of vinylpropylene carbonates with *N-*sulfonyl-1,2,3-triazoles was catalyzed by Pd(0) and Rh(II) at 60°C to form 9-membered monocyclic *N,O*-heterocycle compounds **17**. Heating of the reaction mixture at 120°C–150°C promoted the Alder-ene rearrangement to form bicyclic hexahydropyranopyrroles **16**.

**Scheme 10:**
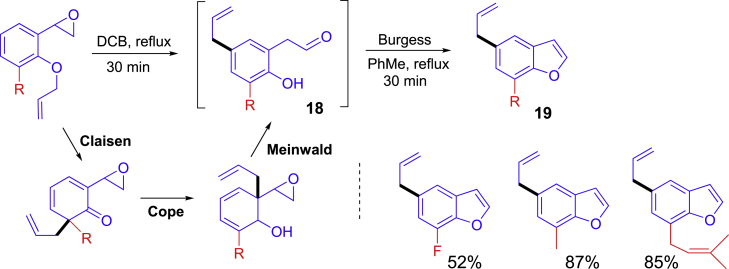
One-pot sigmatropic rearrangement and dehydrative benzofuran formation reactions

Shown in Scheme 10 is an innovative one-pot and two-step synthesis involving thermal Claisen, Cope, and Meinwald rearrangement reactions followed by a Burgess reagent-promoted dehydrative benzofuran formation reaction (Song et al., 2020). Special starting materials bearing epoxide and allyl ether moiety were refluxed in 1,2-dichlorobenzene (DCB) for cascade sigmatropic rearrangement reactions to form intermediate **18**. Switch reaction solvent to toluene and addition of the Burgess reagent for the dehydration reaction afforded substituted benzofurans **19**.

### Multicomponent reaction-involved OPSS

**Scheme 11:**
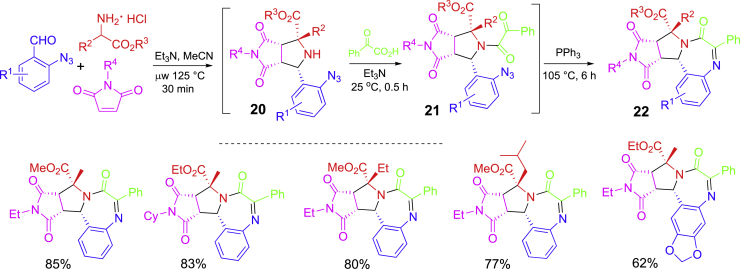
MCR-initiated one-pot synthesis of tetrahydro-pyrrolobenzodiazepinones

A single-step MCR reaction is easy to perform. However, reported numbers of MCRs are limited. Performing post-MCR is a good strategy to increase the structural diversity and also generate new scaffolds (Zhi et al., 2019; Ghashghaei et al., 2019). A one-pot synthesis involving a three-component [3 + 2] cycloaddition followed by amide coupling and aza-Wittig reactions is shown in Scheme 11 (Ma et al., 2019a). In this three-step synthesis, the [3 + 2] cycloaddition was performed as a three-component reaction of 2-azidobenzaldehydes, amino esters, and maleimides under microwave heating to give adducts **20** diastereoselectively. The reaction mixtures were subjected to amide coupling with phenylglyoxylic acid at room temp for **21** and then cyclative aza-Wittig reaction with PPh_3_ at 105°C to afford tetrahydro-pyrrolobenzodiazepinones **22**. Green chemistry metrics analysis indicated that this one-pot synthesis has favorable atom economy, carbon efficiency, mass productivity, and solvent and water intensity.

**Scheme 12:**
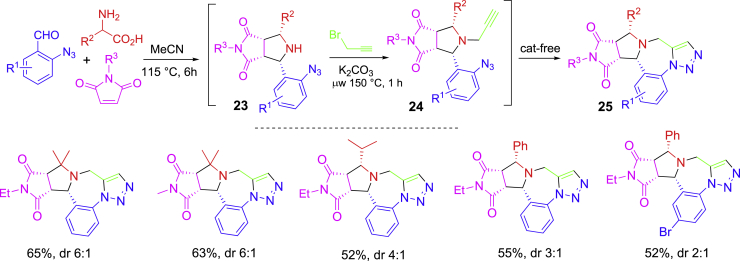
MCR-initiated one-pot synthesis of triazole-fused polycyclic compounds

In another three-component [3 + 2] cycloaddition-initiated synthesis using 2-azidobenzaldehydes, the adducts were used for *N*-propargylation and click reaction to form triazole-fused polycyclic compounds (Scheme 12) (Ma et al., 2019b). Amino acids were used for decarboxylative [3 + 2] cycloaddition with 2-azidobenzaldehydes and maleimides by heating in a sealed tube at 110°C for 6 h. Adducts **23** were used for K_2_CO_3_-promoted *N*-propargylation for **24** and intramolecular triazole formation reactions to give products **25**. It is worth noting that the last step click reaction was spontaneous, no Cu-catalyst was required.

**Scheme 13:**
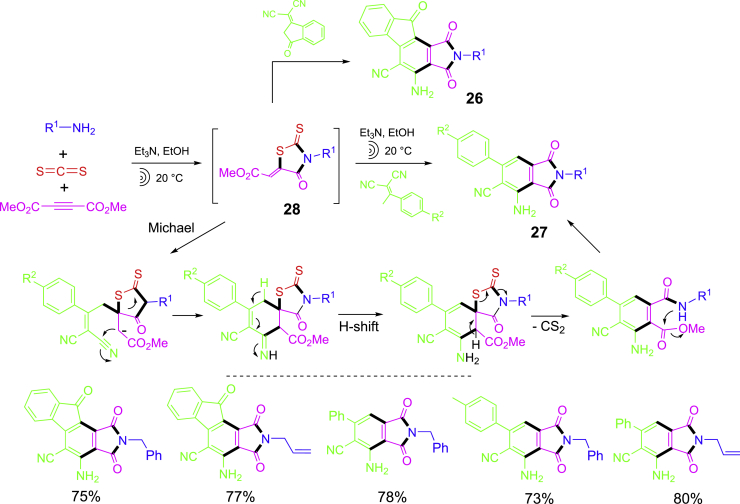
One-pot synthesis of phthalimide-containing scaffolds

An MCR-initiated one-pot synthesis of two phthalimide-containing scaffolds **26** and **27** is shown in Scheme 13 (Alizadeh et al., 2021). A three-component reaction of benzylamine, CS_2_, and dimethyl acetylendicarboxylate afforded 2-thioxothiazolidin-4-one intermediates **28** which were treated with two different malononitriles to afford products **26** and **27**, respectively. Both the MCR and phthalimide formation reactions were conducted under sonication. The second step reaction has a series of transformations including Michael addition, cyclization, elimination of carbon disulfide, and acyl substitution to afford product scaffold.

### Transition metal-catalyzed reactions

Transition metal (TM)-catalyzed reactions are well established and probably still more popular than that of under organocatalysis and biocatalysis. There are many reviews on catalytic one-pot reactions (Vlaar et al., 2011; Afewerki and Cordova, 2016; Szőllősi, 2018) and multicatalysis (Pellissier, 2020b; Martínez et al., 2021). However, the number on catalytic OPSS is very limited, especially for TM-catalyzed reactions due to the catalysts are general sensitive to moisture and air, and with limited scope on substrates. The systems are more complicated in the cases of asymmetric synthesis involving specially designed ligands. A general practice in the development of a TM-catalyzed OPSS is to carry the catalytic reaction prior to other reactions.

**Scheme 14:**
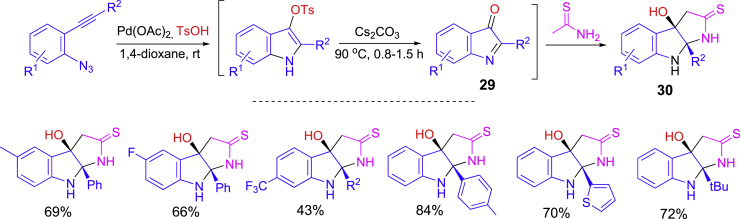
Pd-catalyzed one-pot synthesis of pyrroloindolines

Shown in Scheme 14 is a one-pot synthesis involving Pd-catalyzed formation of indolones followed by second annulation to form pyrroloindoline ring system (Li et al., 2021). The reaction of 1-azido-2-(phenylethynyl)benzenes with TsOH under the catalysis of Pd(OAc)_2_ at room temperature followed by the treatment with CsCO_3_ at 90°C gave **29**. The synthesis was finished by the addition of ethanethioamide for annulation to give pyrroloindoline products **30**.

**Scheme 15:**
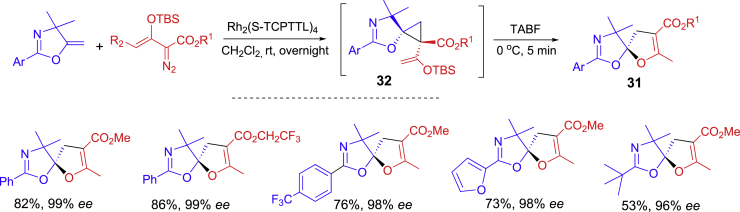
Rh-Catalyzed one-pot asymmetric synthesis of spiroketals

One-pot asymmetric synthesis of spiroketals **31** has been accomplished through Rh-catalyzed cyclopropanation and TABF-promoted ring expansion reactions (Scheme 15) (Dong et al., 2021). A variety of 4,4-dimethyl-methlenedihydrooxazoles and enoldiazoacetates were used for an asymmetric cyclopropanation reaction under the catalysis of Rh_2_(S-TCPTTL)_4_ at room temp for overnight. Intermediates **32** were treated with TABF at 0°C for 5 min for a quick rearrangement reaction to form chiral heterobicyclic spiroketals with high enantioselectivity.

### Organocatalytic asymmetric synthesis

**Scheme 16:**
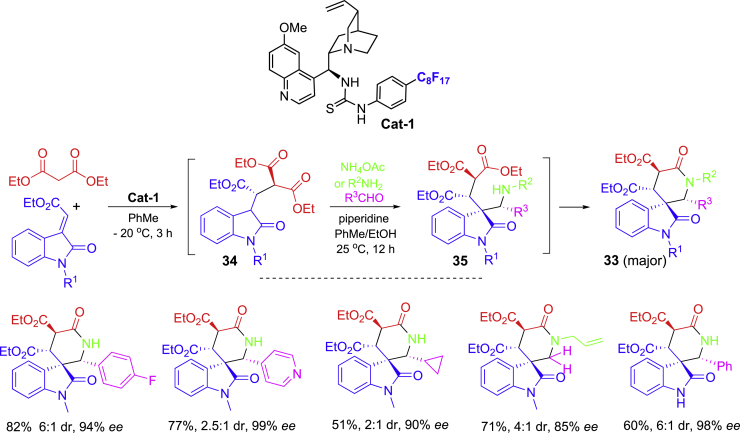
One-pot two-step organocatalytic synthesis of spirooxindoles

Organocatalytic reactions could be performed under mild conditions which make them easier to be developed as OPSS (Chanda and Zhao, 2018; Albrecht et al., 2011). A one-pot and two-step synthesis involving Michael/Mannich/cyclization reactions has been developed for the synthesis of spirooxindoles **33** bearing multiple stereogenic centers (Scheme 16) (Huang et al., 2015). The Michael addition of 1,3-diesters to olefinic oxindoles under the catalysis of recoverable cinchona-based catalyst **Cat-1** for **34** followed by the Mannich reaction with aldehydes and NH_4_OAc or secondary amines for **35** and sequential lactamization afforded spiro-oxindole/*δ*-lactam products **33** with four contiguous stereogenic centers on the newly formed lactam ring. Products have high ee values, but the dr are more substrate dependent.

**Scheme 17:**
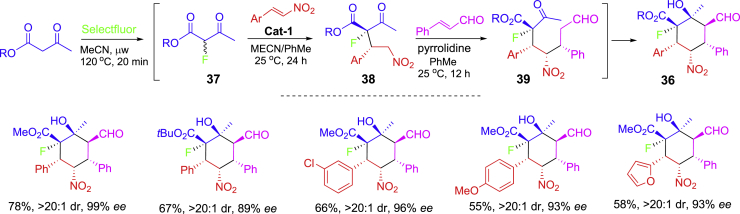
One-pot three-step asymmetric synthesis of substituted cyclohexanols

Recyclable **Cat-1** was used in another one-pot asymmetrical synthesis of biologically interesting cyclohexanols **36.** It involves sequential fluorination/Michael/Michael/aldol reactions (Scheme 17) (Huang et al., 2017). The fluorination of *β*-ketoesters with Selectfluor gave racemic-fluorinated *β*-ketoesters which were used for Michael addition for **37** under the catalysis of **Cat-1** for **38** followed by second Michael addition for **39**, and finally intramolecular aldol reaction to give the products **36** bearing six contiguous stereogenic centers including a fluorinated tertiary carbon. The Michael addition of racemic-fluorinated *β*-ketoesters **37** afforded **38** as the major diastereomers which underwent second Michael addition to form **39** followed by cyclative aldol addition to give products **36** which were stabilized by the intramolecular H-bonding to avoid dehydration.

**Scheme 18:**
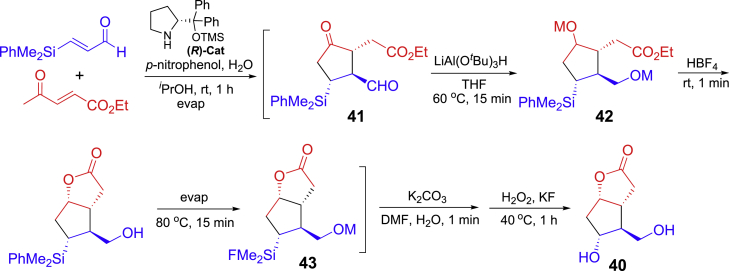
Organocatalysis-initiated one-pot asymmetric synthesis of Corey lactone

Corey lactone is an asymmetric bicyclic compound which is a versatile intermediate for the synthesis of prostaglandin hormones. A multistep synthesis initiated with the diphenylprolinol silyl ether-catalyzed Michael/Michael reactions of 3-(dimethylphenylsilyl) propenal and ethyl 4-oxo-2-pentenoate is shown in Scheme 18 (Umekubo et al., 2020). Intermediate **40** was treated with 3.5 equiv of LiAl(O^*t*^Bu)_3_H to reduce ketone and aldehyde carbonyls for **41** followed by addition of HBF_4_ to form dihydroxy ester which immediately cyclized to give lactone **42** and then **43** after evaporation of THF solvent. Neutralization of the reaction mixture with K_2_CO_3_ followed by oxidative alkyl silyl removal with H_2_O_2_ and KF afforded the Corey lactone. This one-pot six-operation-step synthesis could be done in 152 min to give the product in 99% *ee*.

### Synthesis of natural products and bioactive molecules

Mother nature is a master of performing highly efficient and yet selective enzymatic multistep synthesis for making complicated molecules in living organisms. Synthetic chemists have accomplished numerous natural product synthesis with the assistance of newly developed techniques (Dückert et al., 2012; Ardkhean et al., 2016; Nicolaou et al., 2003) including biosynthesis (Riva and Fessner, 2014). But non-biocatalytic OPSS for total synthesis is still a dream to be fully realized. Some successes have been achieved on the synthesis of key intermediates in natural product total synthesis (Pellissier, 2020b).

**Scheme 19:**
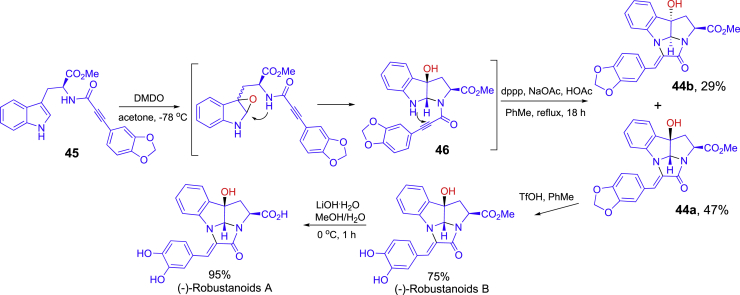
One-pot synthesis of intermediate **X** for (−)-robustanoids B and A

Highlighted in Scheme 19 is a one-pot synthesis trigged by 3,3-dimethyldioxirane (DMDO) epoxidation in the synthesis of **44a** which is an intermediate for (−)-robustanoids B and A (Scheme 18) (Liu and Huang, 2019). DMDO epoxidation of **45** in acetone at −78°C was followed by bis(1-3-diphenylphosphino)propane (dppp)-promoted cyclization to form **44a** and **44b** as a mixture of diastereomers. Isolated diastereomer 5 was readily converted to for (−)-robustanoids B and A.

**Scheme 20:**
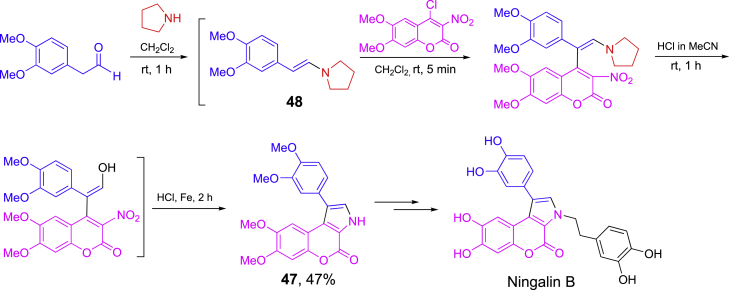
One-pot synthesis of intermediate **47** for ningalin B

A one-pot three-step synthesis of intermediates **47** in the total synthesis ningalin B is shown in Scheme 20 (Wu et al., 2019). Condensation of 2-(3,4-dimethoxyphenyl) acetaldehyde and pyrrolidine afforded enamine **48** which was reacted with 4-chloro-3-nitrocoumarin followed by enamine hydrolysis and NO_2_ reduction with Fe under acidic condition to form chromenopyrrol-4-one **47**. Additional two-step reactions from **47** finished the total synthesis of ningalin B. In the one-pot synthesis, the solvent was switched from CH_2_Cl_2_ for the first two steps to MeCN at the third step.

**Scheme 21:**
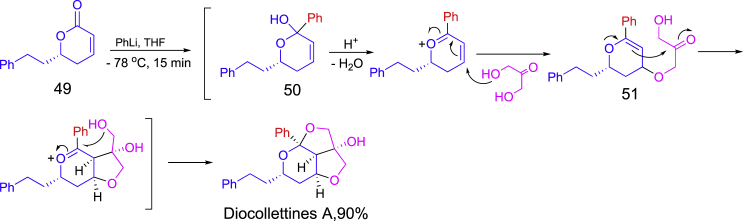
One-pot synthesis in the preparation of diocollettines A

Diocollettines A has a unique oxygen-containing tricyclic core. One-pot synthesis was applied to the last steps of the total synthesis of diocollettines A (Scheme 21) (Kawamoto et al., 2019). Enantiopure compound **49** was treated with PhLi at −78°C to form transient intermediate **50** which then reacted with 1,3-dihydroxyacetone (DHA) under basic conditions to give the tricyclic product diastereoselectively through the processes of *trans*-selective oxa-Michael addition for **51**, aldol-type cyclization, and intramolecular acetalization.

**Scheme 22:**
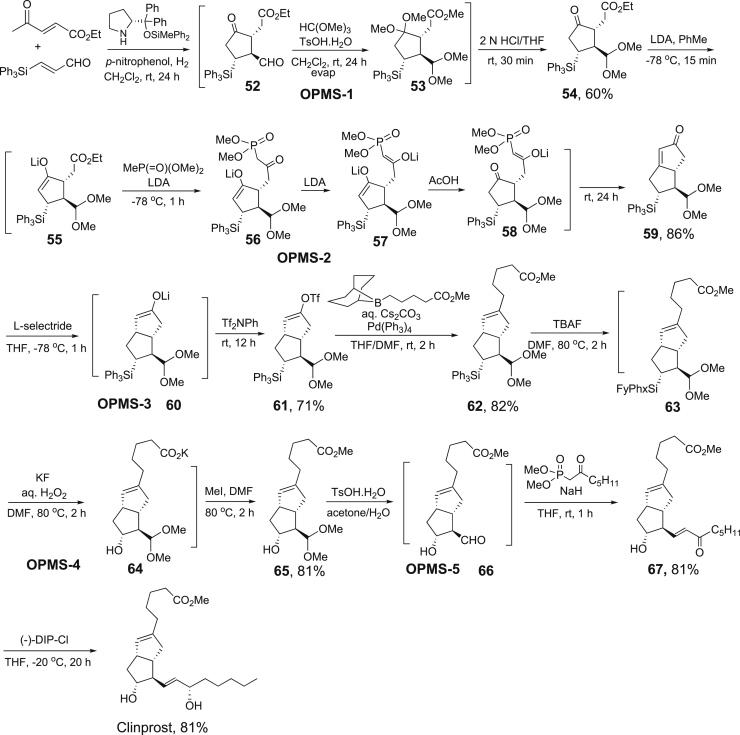
Total synthesis of clinprost involving five steps of OPSS

Clinprost is a bicyclo[3.3.0]octenone core with four stereogenic centers. Extensive effort on the integration of one-pot synthesis was invested in a recently reported total synthesis of clinprost (Scheme 22) (Umekubo and Hayashi, 2020). Five OPSS were incorporated into a 17-step synthesis. The first OPSS was initiated with the organocatalyst-promoted asymmetric [3 + 2] cycloaddition of ethyl 4-oxo-2-pentenoate with 3-(triphenylsilyl)propenal to form **52** which was subjected to double carbonyl group acetalization to form **53** and then selective hydrolysis to finish the one-pot synthesis of **54** in 60%. This intermediate was used for a new one-pot synthesis treated with LDA to form enolate **55** for Claisen reaction with dimethylmethylphosphonate to afford *β*-keto phosphonate **56** which was quickly converted to dianion **57**. Addition of AcOH selectively regenerated a keto group in **58** to afford bicyclo[3.3.0]octenone **59** as a Horner−Wadsworth−Emmons reaction product in 86%. In the third one-pot two-step synthesis, **59** was treated with L-selectride to generate enolate **60** which was trapped with Tf_2_NPh to afford **61** in 82%. After the Suzuki−Miyaura coupling reaction to convert **61** to **62**, the fourth OPSS took place by treated **62** with TBAF for **63** and then with KF for **64** followed by addition of MI to form methyl ester **24** in 81%. The last OPSS was to treat **65** with TsOH hydrolysis the acetal to form **66** for Horner−Wadsworth−Emmons reaction to afford **67** in 81%. Asymmetric reduction of keto group with (−)-DIP-Cl afforded clinprost in 85% yield. This is an excellent example of implement OPSS strategy in the total synthesis. If all the reaction steps were conducted in one-pot, this could be a dream sequence in asymmetric total synthesis of a natural product.

**Scheme 23:**
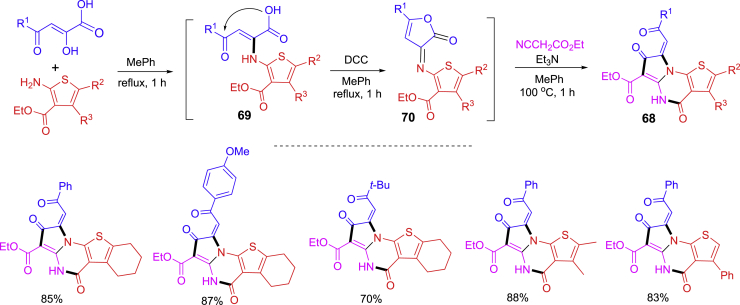
One-pot synthesis of bioactive thieno[3,2-*e*]pyrrolo[1,2-*a*]pyrimidines

Thieno[3,2-*e*]pyrrolo[1,2-*a*]pyrimidines **68** highlighted in Scheme 23 are biologically interesting compounds with potential poly(ADP-ribose) polymerase-1 (PARP-1) inhibitory activity (Shipilovskikh and Rubtsov, 2019). An OPSS protocol has been developed start from the reaction of Gevald’s aminothiophene and 2-hydroxy-4-oxobut-2-enoic acid to form 2-(2-tiennyl)amino-4-oxobut-2-enoic acid **69** which underwent dicyclohexylcarbodiimide (DCC)-promoted dehydrative cyclization to yield furanone **70**. It was treated with cyanoacetic ester and Et_3_N under heating the reaction mixture at 100°C for 1 h to afford products **68.** Toluene was the only solvent used in this one-pot three-step synthesis.

### Summary

One of the aims of this perspective article is to define the one-pot stepwise synthesis (OPSS) and to differentiate it from the cascade reaction and multicomponent reaction (MCR). Even these three one-pot reactions share some common green chemistry advantages such as low cost, high efficiency, resource saving, and minimal waste disposal. The OPSS could be operational more flexible and transformational more feasible than other two methods. Since there is no focused review on OPSS, this paper uses selected examples to demonstrate the recent development of OPSS in the synthesis of biologically interesting heterocyclic compounds, asymmetric molecules, and natural products through cyclization, cycloaddition, and rearrangement reactions.

I like to offer my personal perspective on the future development of OPSS. Design and discovery of new OPSS could have great potential in the areas such as catalytic reactions involving dual or multiple catalysts. The catalysts could be transition metals, enzymes, and organic molecules, or a mixture use of these three classes of catalysts. Conducting an MCR followed by post-condensation modifications is a good direction for OPSS for making complicated structures. In addition to the development of new OPPS, reengineer reported multistep synthesis could be important, especially in the synthetic optimization for drug molecules and natural products. More success on OPSS could be achieved by performing following practices: 1) streamlining the synthetic process and maximizing the conversion for each step, 2) balancing substrate scope, functional group tolerance, and reaction selectivity, 3) incorporating more reaction steps for higher efficiency in making complicated structures, and 4) integrating different kind of reactions (such as ionic and radical reactions) or using different catalysts (such as organo, metal, and photoredox catalysts) which are hard to achieve in MCRs and cascade reactions. The introduction of new software and programs for AI-assisted synthesis and computational molding is increasingly important for the designing of new OPSS. Interdisciplinary effort and systematic approach to incorporate new synthetic techniques such as flow chemistry, mechanochemistry, and photoredox and biocatalysis could sets up new platforms for the development of OPSS (Constable, 2021). Promoting the reactions in green solvents such as water, ionic liquids, and supercritical fluid (Horváth, 2008) and using alternative energy such as microwave, light, sonication, and electricity (Robertson et al., 2019) also provides new avenues for OPSS in the synthesis of small molecules with biological interests.

## References

[bib1] Abou-Shehada S., Mampuys P., Maes B.U.W., Clark J.H., Summerton L. (2017). An evaluation of credentials of a multicomponent reaction for the synthesis of isothioureas through the use of a holistic CHEM21 green metrics toolkit. Green Chem..

[bib2] Afewerki S., Cordova A. (2016). Combinations of aminocatalysts and metal catalysts: a powerful cooperative approach in selective organic synthesis. Chem. Rev..

[bib3] Albrecht Ł., Jiang H., Jørgensen K.A. (2011). A simple recipe for sophisticated cocktails: organocatalytic one-pot reactions – concept, nomenclature, and future perspectives. Angew. Chem. Int. Ed. Engl..

[bib4] Alizadeh A., Farajpour B., Knedel T.-O., Janiak C. (2021). Synthesis of substituted phthalimides via ultrasound-promoted one-pot multicomponent reaction. J. Org. Chem..

[bib5] Anastas P., Eghbali N. (2010). Green chemistry: principles and practice. Chem. Soc. Rev..

[bib6] Ardkhean R., Caputo D.F.J., Morrow S.M., Shi H., Xiong Y., Anderson E.A. (2016). Cascade polycyclizations in natural product synthesis. Chem. Soc. Rev..

[bib7] Broadwater S.J., Roth S.L., Price K.E., Kobašlija M., McQuade D.T. (2005). One-pot multi-step synthesis: a challenge spawning innovation. Org. Biomol. Chem..

[bib8] Bruggink A., Schoevaart R., Kieboom T. (2003). Concepts of nature in organic synthesis: cascade catalysis and multistep conversions in concert. Org. Process Res. Dev..

[bib9] Chanda T., Zhao J.C. (2018). Recent progress in organocatalytic asymmetric domino transformations. Adv. Synth. Catal..

[bib10] Cioc R.C., Ruijter E., Orru R.V.A. (2014). Multicomponent reactions: advanced tools for sustainable organic synthesis. Green Chem..

[bib11] Clark J., Macquarrie J. (2002).

[bib12] Clarke P.A., Santos S., Martin W.H.C. (2007). Combining pot, atom and step economy (PASE) in organic synthesis. Synthesis of tetrahydropyran-4-ones. Green Chem..

[bib13] Climent M.J., Corma A., Iborra S., Mifsud M., Velty A. (2010). New one-pot multistep process with multifunctional catalysts: decreasing the E factor in the synthesis of fine chemicals. Green Chem..

[bib14] Constable D.J.C. (2021). Green and sustainable chemistry – the casefor a systems-based, interdisciplinary approach. iScience.

[bib15] Constable D.J.C., Jiménez-González C., Green Metrics Anastas P. (2018).

[bib16] Curran D.P., Rakiewicz D.M. (1985). Tandem radical approach to linear condensed cyclopentanoids. Total synthesis of (+/-)-hirsutene. J. Am. Chem. Soc..

[bib17] Denard C.A., Hartwig J.F., Zhao H. (2013). Multistep one-pot reactions combining biocatalysts and chemical catalysts for asymmetric synthesis. ACS Catal..

[bib18] Domling A., Wang W., Wang K. (2012). Chemistry and biology of multicomponent reactions. Chem. Rev..

[bib19] Dong K., Gurung R., Xu X., Doyle M.P. (2021). Enantioselective catalytic cyclopropanation-rearrangement approach to chiral spiroketals. Org. Lett..

[bib20] Dückert H., Pries V., Khedkar V., Menninger S., Bruss H., Bird A.W., Maliga Z., Brockmeyer A., Janning P., Hyman A. (2012). Natural product–inspired cascade synthesis yields modulators of centrosome integrity. Nat. Chem. Biol..

[bib21] Espeel P., Du Prez F.E. (2015). One-pot multi-step reactions based on thiolactone chemistry: a powerful synthetic tool in polymer science. Eur. Polym. J..

[bib22] Gabriele B., Mancuso R., Veltri L., Ziccarelli I., Della Ca' N. (2019). Palladium-catalyzed double cyclization processes leading to polycyclic heterocycles: recent advances. Eur. J. Org Chem..

[bib23] Ganesan S., Sarangapani M., Doble M. (2020). An expedient, one-pot, stepwise sequential approach for the regioselective synthesis of pyrazolines. J. Chem. Res..

[bib24] Ghashghaei O., Seghetti F., Lavilla R. (2019). Selectivity in multiple multicomponent reactions: types and synthetic applications. Beilstein J. Org. Chem..

[bib25] Groger H., Hummel W. (2014). Combining the ‘two worlds’ of chemocatalysis and biocatalysis towards multi-step one-pot processes in aqueous media. Curr. Opin. Chem. Biol..

[bib26] Hayashi Y. (2016). Pot economy and one-pot synthesis. Chem. Sci..

[bib27] Hayashi Y. (2021). Time and pot economy in total synthesis. Acc. Chem. Res..

[bib28] Heravi M.M., Zadsirjan V. (2020).

[bib29] Horváth I.T. (2008). Solvents from nature. Green Chem..

[bib30] Howard J.L., Nicholson W., Sagatov Y., Browne D.L. (2017). One-pot multistep mechanochemical synthesis of fluorinated pyrazolones. Beilstein J. Org. Chem..

[bib31] Hoxha S., Kalaitzakis D., Bosveli A., Montagnon T., Vassilikogiannakis G. (2021). One-pot transformation of furans into 1-azaspirocyclic alkaloid frameworks induced by visible light. Org. Lett..

[bib32] Huang H.-M., Garduño-Castro M.H., Morrill C., Procter D.J. (2019). Catalytic cascade reactions by radical relay. Chem. Soc. Rev..

[bib33] Huang X., Liu M., Jasinski J.P., Peng B., Zhang W. (2017). Recyclable organocatalysts for a one-pot asymmetric synthesis of 2-fluorocyclohexanols bearing six contiguous stereocenters. Adv. Synth. Catal..

[bib34] Huang X., Pham K., Yi W., Zhang X., Clamens C., Hyatt J.H., Jasinsk J.P., Tayvah U., Zhang W. (2015). Recyclable organocatalyst-promoted one-pot asymmetric synthesis of spirooxindoles bearing multiple stereogenic centers. Adv. Synth. Catal..

[bib35] Jones A.C., May J.A., Sarpong R., Stoltz B.M. (2014). Toward a symphony of reactivity: cascades involving catalysis and sigmatropic rearrangements. Angew. Chem. Int. Ed. Engl..

[bib36] Kawamoto M., Sato S., Enomoto M., Ogura Y., Kuwahara S. (2019). Total synthesis of Diocollettines A via an acid-promoted oxa-Michael-Aldol-acetalization cascade. Org. Lett..

[bib37] Kumar R., Asthana M., Singh R.M. (2017). Pd-catalyzed one-pot stepwise synthesis of benzo[b] [1, 6]naphthyridines from 2-Chloroquinoline-3-carbonitriles using sulfur and amines as nucleophiles. J. Org. Chem..

[bib38] Lee K.R., Ahn S., Lee S.G. (2021). Synergistic Pd(0)/Rh(II) dual catalytic [6 + 3] dipolar cycloaddition for the synthesis of monocyclic nine-membered *N, O*-heterocycles and their alder-ene rearrangement to fused bicyclic compounds. Org. Lett..

[bib39] Ley S.V., Baxendale I.R., Bream R.N., Jackson P.S., Leach A.G., Longbottom D.A., Nesi M., Scott J.S., Storer R.I., Taylor S.J. (2000). Multi-step organic synthesis using solid-supported reagents and scavengers: a new paradigm in chemical library generation. J. Chem. Soc. Perkin 1.

[bib40] Li P., Yang F., Hu G., Zhang X. (2021). Palladium-catalyzed one-pot synthesis of pyrroloindolines from 2-alkynyl arylazides and thioacetamides. J. Org. Chem..

[bib41] Liu Z.-J., Huang P.-Q. (2019). Biomimetic enantioselective total synthesis of (-)-robustanoids A and B and analogues. J. Org. Chem..

[bib42] Liu S., Xu L., Wei Y. (2019). One-pot, multistep reactions for the modular synthesis of *N, N′*-Diarylindazol-3-ones. J. Org. Chem..

[bib43] Ma X., Zhang X., Awad J.M., Xie G., Qiu W., Zhang W. (2019). One-pot synthesis of tetrahydro-pyrrolobenzodiazepines and tetrahydro-pyrrolobenzodiazepinones through sequential 1, 3-dipolar cycloaddition/N-alkylation (N-acylation)/Staudinger/aza-Wittig reactions. Green Chem..

[bib44] Ma X., Zhang X., Qiu W., Zhang W., Wan B., Evans J., Zhang W. (2019). One-pot synthesis of triazolobenzodiazepines through decarboxylative [3 + 2] cycloaddition of nonstabilized azomethine ylides and Cu-free click reactions. Molecules.

[bib45] Mao X., Tong T., Fan S., Fang L., Wu J., Wang X., Kang H., Lv X. (2017). One-pot synthesis of thiazino[2, 3, 4-hi]indole derivatives through a tandem oxidative coupling/heteroannulation process. Chem. Commun..

[bib46] Martínez S., Veth L., Lainer B., Dydio P. (2021). Challenges and opportunities in multicatalysis. ACS Catal..

[bib47] McElroy C.R., Constantinou A., Jones L.C., Summerton L., Clark J.H. (2015). Towards a holistic approach to metrics for the 21st century pharmaceutical industry. Green Chem..

[bib48] Merrifield B. (1986). Solid phase synthesis. Science.

[bib49] Muthengi A., Zhang X., Dhawan G., Zhang W., Corsini F., Zhang W. (2018). Sequential (3 + 2) cycloaddition and (5 + n) annulation for modular synthesis of dihydrobenzoxazines, tetrahydrobenzoxazepines and tetrahydrobenzoxazocines. Green Chem..

[bib50] Nicolaou K.C., Montagnon T., Snyder S.A. (2003). Tandem reactions, cascade sequences, and biomimetic strategies in total synthesis. Chem. Commun..

[bib51] Nikolić A.M., Živković F., Selaković Ž., Wipf P., Opsenica I.M. (2020). One-pot two-step synthesis of isochromene-fused CF_3_-substituted pyrazoles. Eur. J. Org. Chem..

[bib52] Pellissier H. (2020). The use of domino reactions for the synthesis of chiral rings. Synthesis.

[bib53] Pellissier H. (2020). Recent developments in enantioselective multicatalyzed tandem reactions. Adv. Synth. Catal..

[bib54] Sears J.E., Boger D.L. (2016). Tandem intramolecular Diels−Alder/1, 3-dipolar cycloaddition cascade of 1, 3, 4-oxadiazoles: initial scope and applications. Acc. Chem. Res..

[bib55] Riva S., Fessner W.-D. (2014). Cascade biocatalysis: integrating stereoselective and environmentally friendly reactions.

[bib56] Robertson J.C., Coote M.L., Bissember A.C. (2019). Synthetic applications of light, electricity, mechanical force and flow. Nat. Rev. Chem.

[bib57] Sheldon R.A. (2018). Metrics of green chemistry and sustainability: past, present, and future. ACS Sustainable Chem. Eng..

[bib58] Shipilovskikh S.A., Rubtsov A.E. (2019). One-pot synthesis of thieno[3, 2-e]pyrrolo[1, 2-a]pyrimidine derivative scaffold: a valuable source of PARP-1 inhibitors. J. Org. Chem..

[bib59] Snyder S.A., Schaumann E. (2016). Science of Synthesis: Applications of Domino Transformations in Organic Synthesis.

[bib60] Song L., Su Q., Lin X., Du Z., Xu H., Ouyang M.-A., Yao H., Tong R. (2020). Cascade Claisen and Meinwald rearrangement for one-pot divergent synthesis of benzofurans and 2*H*-chromenes. Org. Lett..

[bib61] Sui Z., An R., Komiyama M., Liang X. (2021). Stepwise strategy for one-pot synthesis of single-stranded DNA rings from multiple short fragments. Chembiochem.

[bib62] Szőllősi G. (2018). Asymmetric one-pot reactions using heterogeneous chemical catalysis: recent steps towards sustainable processes. Catal. Sci. Technol..

[bib63] Tietze L.F., Brasche G., Gericke K.M. (2006).

[bib64] Trost B.M. (2002). On inventing reactions for atom economy. Acc. Chem. Res..

[bib65] Umekubo N., Hayashi Y. (2020). Pot-economical total synthesis of Clinprost. Org. Lett..

[bib66] Umekubo N., Suga Y., Hayashi Y. (2020). Pot and time economies in the total synthesis of Corey lactone. Chem. Sci..

[bib67] Vaccaro L. (2017). Sustainable flow chemistry: methods and applications.

[bib68] Vlaar T., Ruijter E., Orru R.V.A. (2011). Recent advances in palladium-catalyzed cascade cyclizations. Adv. Synth. Catal..

[bib69] Wang D., Sun J., Liu R.-Z., Wang Y., Yan C.-G. (2021). Diastereoselective synthesis of tetrahydrospiro[carbazole-1, 3’-indolines] *via* an InBr_3_-catalyzed domino Diels-Alder reaction. J. Org. Chem..

[bib70] Wender P.A. (2013). Toward the ideal synthesis and transformative therapies: the roles of step economy and function oriented synthesis. Tetrahedron.

[bib71] Wu C.-K., Weng Z., Yang D.-Y. (2019). One-pot construction of 1-phenylchromeno[3, 4-*b*]pyrrol-4(3H)-one: applicationto total synthesis of ningalin B and a pyrrolocoumarin-based electrochromic switch. Org. Lett..

[bib72] Yoon S.H., Kim S.J., Kim I. (2020). One-pot four-component coupling approach to polyheterocycles: 6H-furo[3, 2-f]pyrrolo[1, 2-d] [1, 4]diazepine. J. Org. Chem..

[bib73] Zhang B., Studer A. (2015). Recent advances in the synthesis of nitrogen heterocycles *via* radical cascade reactions using isonitriles as radical acceptors. Chem. Soc. Rev..

[bib74] Zhang W., Cue B.W. (2018). Green techniques for organic synthesis and medicinal chemistry.

[bib75] Zhang W., Yi W.-B., Sharma S.K., Rajasthan J. (2019). SpringerBriefs in Green Chemistry for Sustainability.

[bib76] Zhang X., Qiu W., Ma X., Evans J., Kaur M., Jasinski J.P., Zhang W. (2018). One-pot double [3 + 2] cycloadditions for diastereoselective synthesis of pyrrolidine-based polycyclic systems. J. Org. Chem..

[bib77] Zheng L., Hua R. (2020). Recent advances in construction of polycyclic natural product scaffolds *via* one-pot reactions involving alkyne annulation. Front. Chem..

[bib78] Zhi S., Ma X., Zhang W. (2019). Consecutive multicomponent reactions for the synthesis of complex molecules. Org. Biomol. Chem..

[bib79] Zhu J., Wang Q., Wang M.-X. (2014).

